# And the Last Shall Be First: Heterochrony and Compensatory Marine Growth in Sea Trout (*Salmo trutta*)

**DOI:** 10.1371/journal.pone.0045528

**Published:** 2012-10-01

**Authors:** Francisco Marco-Rius, Pablo Caballero, Paloma Morán, Carlos Garcia de Leaniz

**Affiliations:** 1 Departamento de Bioquímica, Genética e Inmunología, Universidad de Vigo, Vigo, Spain; 2 Consellería de Medio Rural, Servizo de Conservación da Natureza, Xunta de Galicia, Pontevedra, Spain; 3 Department of BioSciences, Swansea University, Swansea, United Kingdom; Oxford Brookes University, United Kingdom

## Abstract

Early juvenile growth is a good indicator of growth later in life in many species because larger than average juveniles tend to have a competitive advantage. However, for migratory species the relationship between juvenile and adult growth remains obscure. We used scale analysis to reconstruct growth trajectories of migratory sea trout (*Salmo trutta*) from six neighbouring populations, and compared the size individuals attained in freshwater (before migration) with their subsequent growth at sea (after migration). We also calculated the coefficient of variation (CV) to examine how much body size varied across populations and life stages. Specifically, we tested the hypothesis that the CV on body size would differ between freshwater and marine environment, perhaps reflecting different trade-offs during ontogeny. Neighbouring sea trout populations differed significantly in time spent at sea and in age-adjusted size of returning adults, but not on size of seaward migration, which was surprisingly uniform and may be indicative of strong selection pressures. The CV on body size decreased significantly over time and was highest during the first 8 months of life (when juvenile mortality is highest) and lowest during the marine phase. Size attained in freshwater was negatively related to growth during the first marine growing season, suggesting the existence of compensatory growth, whereby individuals that grow poorly in freshwater are able to catch up later at sea. Analysis of 61 datasets indicates that negative or no associations between pre- and post-migratory growth are common amongst migratory salmonids. We suggest that despite a widespread selective advantage of large body size in freshwater, freshwater growth is a poor predictor of final body size amongst migratory fish because selection may favour growth heterochrony during transitions to a novel environment, and marine compensatory growth may negate any initial size advantage acquired in freshwater.

## Introduction

Many animals pass through some migratory stage during their lives [1]–[2], typically in relation to feeding or reproduction. Migrations are thought to maximise age-specific fecundity and the probability of surviving from one breeding season to the next [3]–[4], and have been interpreted as a response to adversity [5].

Migrations are energetically costly and a trade off may be expected to exist between the costs of migrations and the fitness benefits accrued by a larger body sizes [1], as well as between predator avoidance and feeding gains [6]. Migrants typically achieve a larger body size than non-migrants, but may also sustain higher mortality rates than resident individuals [7]–[8]. Such trade-offs between growth and mortality are common in many species and can reflect a balance between foraging and predation risk, growth and maturation, and growth and resistance to diseases, amongst others [9]. These may result in individuals achieving similar fitness, despite having grown at widely different rates [10].

Amongst anadromous salmonids, which must migrate between very different freshwater and marine environments, the risk of predation increases at sea [11] and a relatively narrow optimum size at migration appears to exist [12]. Yet, size at migration (smolt size) and growth during the first marine season (post smolt growth) are perhaps the traits that differ the most amongst populations [7], [13]–[14], probably because homing behaviour tends to result in geographical isolation and locally adapted populations [15]. Maturation schedules also tend to differ greatly amongst populations [16], even among fish inhabiting neighbouring rivers [13], [17] suggesting the existence of different and spatially localised trade-offs.

Field studies have revealed contrasting selection pressures for body size of anadromous salmonids in freshwater and marine environments [18]–[21] suggesting the existence of different trade-offs in rivers and sea. Yet, the relationship between pre-migratory growth in freshwater (i.e. smolt size) and post-migratory growth in the sea (i.e. post-smolt growth) is not clear. There appear to be as many studies reporting a negative relationship between smolt length and marine growth [16], [22]–[24] as there are studies reporting a positive or no relationship [25]–[27]. This suggests that there can be considerable variation in the way individuals adapt to environmental change during their transition from freshwater to marine environments.

Here we used scale image analysis to reconstruct individual growth trajectories of migratory brown (sea trout, *Salmo trutta*) in order to examine the relationship between smolt size and post smolt growth in six neighbouring populations. As selection can act strongly on body size and size-related traits in juvenile salmonids [15], we used the coefficient of variation on body size (CV) in order to quantify the extent of phenotypic variation [28] across different life stages. Specifically, we tested the hypothesis that the coefficient of variation on body size of migratory trout would differ between the freshwater and marine environment, perhaps reflecting different trade-offs during ontogeny [22]–[23]. Furthermore, because selection in fishes tends to be strongest during the early juvenile stages - when mortality is highest but when body size is also smallest [29]–[30] - we also expected to find a negative relationship between developmental stage and the extent of individual variation in body size.

## Methods

### Study Populations

**Figure 1:**
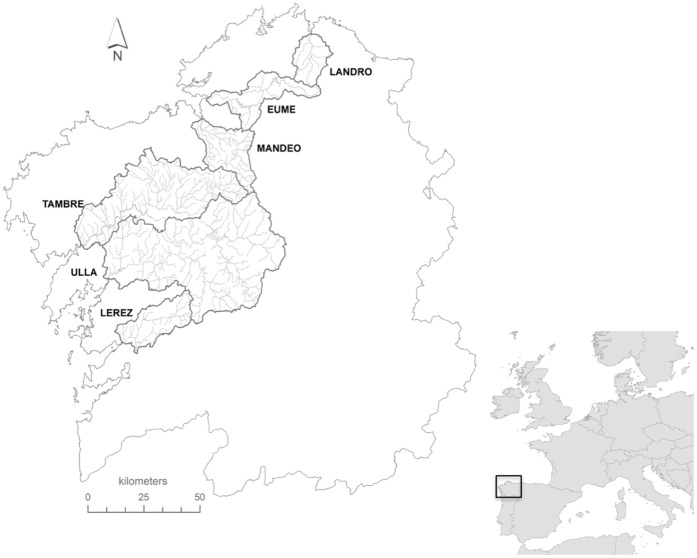
Study sea trout (*Salmo trutta*) populations in Galicia, NW Spain.

Migratory sea trout were caught between August and October 2002 on their returning migration by government officials in upstream traps or by angling by licensed sport fishermen in six neighbouring rivers in NW Spain (Fig. 1). Study populations differed in physical as well as in key demographic parameters, including population abundance (as inferred from rod and line caches), age and body size, and expected survival (as inferred from incidence of multiple spawners and maximum longevity; Table 1). Upstream migrants were assumed to have been caught on their river of origin as these populations had shown isolation by distance and restricted gene flow, which are suggestive of strong homing behaviour [31].

**Table 1:** Physical and demographic parameters (means ± SE) of study populations of migratory brown trout (*Salmo trutta*).

River	Mean annual flow (m^3^/s)	Accessible reach(Km)	Watershed(Km^2^)	Estuary(Km^2^)	Stream order	Rod and line catch	Max. bodysize(mm)	Mean smoltsize(mm)	Meansmoltage(yr)	Mean age at return (yr)	Max. longevity (yr)
Eume	19.3	13.3	470.2	64	5	537	440	224±5.8	2.12±0.08	3.69±0.14	5
Landro	7.2	22.6	269.6	10	5	113	450	212±7.5	2.20±0.11	3.35±0.13	4
Mandeo	14.2	20.7	456.9	64	5	479	540	210±7.1	2.14±0.09	4.10±0.19	6
Lerez	21.3	25.1	449.5	99	5	6	490	174±9.1	2.04±0.13	3.43±0.15	5
Tambre	54.2	5.7	1530	133	6	356	550	205±11.4	2.30±0.12	3.55±0.11	4
Ulla	79.7	102.3	2803.6	238	6	351	480	220±12.4	2.12±0.12	3.33±0.13	4

### Ethics Statement

Collection of scale samples was carried out by fisheries staff of the Regional Government of Galicia (Wildlife Service) using a non-intrusive procedure and according to current Spanish Regulations. No specific permits were required for the described field studies, and these did not involve endangered or protected species.

### Scale Analysis and Growth Profiles

Scales of 30 individuals per river were stored dry in paper envelopes, along with information on their body size (fork length, mm). Between three and five scales with a clear (non–regenerated) nucleus were selected per individual to prevent bias due to loss of growth rings [32]. Acetate impressions were made with the aid of a pressure roller and the resulting impressions were then scanned with a Minolta MS 6000 microfilm scanner at 23–50× magnifications and saved as high resolution TIFF images as in [33].

The software *Image-J* v. 1.4.1 [34] was employed to digitize the position of each growth ring (circuli), to identify the annual growth rings (annuli), and to measure the inter-circuli spacing along the 360° scale axis with reference to a calibrated scale bar in order to derive measures of scale growth [35]. The freshwater and marine ages were determined based on the number of annuli [36], and the points of entry of smolts into the sea (beginning of marine phase) and end of the first marine growing season (post-smolt growth, PSG) were noted [16]. Twenty three finnocks (individuals which had returned to freshwater before completing one full winter at sea [37]) were excluded from analysis as these provided no comparable data on post-smolt growth.

Individual growth profiles were obtained by plotting circuli number against scale size at four key life stages: (a) first freshwater winter, (b) moment of entry into the sea, (c) end of first marine growing season, and (d) return of adults into freshwater from the sea. Ordinary Least Squares (OLS) regression was then used to determine scale growth slopes, measured between the scale focus and the scale edge [38].

### Reliability of Scale Analysis

A paired *t*-test was used to assess non-random deviations in scale radii between the original scales and their acetate impressions (n = 30) in order to quantify potential bias in scale measurements arising from pressure from the hand roller. To ascertain the precision of the scale analysis, we estimated the repeatability of the point of entry into the sea and of the end of the first marine growing season by measuring the scales of 30 individuals twice in a double blind fashion and calculating the intra-class correlation coefficient (α-Cronbach) as per [33]. The Pearson correlation coefficient was used to evaluate the strength of the association between scale radius and body size of fish in each river. The coefficients of variation (CV = SD/mean) were then examined to compare the precision of body size and scale measurements. Precision in scale measurements (0.01 mm; CV = 13.9%) was better than that of body size measurements (cm; CV = 15.3%), and the former was therefore preferred to examine growth variation among migratory trout.

In order to evaluate if the relationship between somatic growth and scale radius changed with age or body size, we tested for homogeneity of slopes in an ANCOVA model [39] using either age (five age classes) or body size (four size quartiles) as covariates. We also checked that the relationship between age and body size (Log_10_) was linear within the limits of this study (*F*_1,126_ = 17.392, *P*<0,001), and not different among rivers (River *F*_5,126_ = 0.323, *P = *0.898; River×Age interaction *F*_5,126_ = 0.060, *P* = 0.998).

### Statistical Analysis

Analyses were carried out using SYSTAT 10.0 and the R software [40]. The R MASS package [41] was used to model variation in post smolt growth (PSG) in relation to smolt size, smolt age and river identity, and the Akaike information criteria (AIC) was used for model selection.

We employed the CV to quantify phenotypic variation in body size [28] among populations and across four different key life stages (first freshwater winter, moment of entry into the sea, first marine growing season, and return as adult to the river). Only individuals that had spent 2 years in freshwater (S2 smolts) were used, as this was the dominant smolt age in the study populations and the number of fish of other smolt ages was low. Approximate 95% confidence limits were constructed by bootstrapping 1,000 replicates, and the Fligner-Killen test (a non-parametric version of Levene’s test which is robust to departures of normality [42]) was used to compare differences in CV among stages of development and among rivers.

We visualized growth reaction norms during the freshwater to marine transition by plotting individual growth trajectories between the moment of entry into the sea (smolt size) and the growth of the first marine growing season (PSG). These trajectories described how individuals responded to environmental change according to population of origin and smolt age. Variation in individual growth trajectories was analysed by repeated measures ANCOVA using river of origin as a fixed factor and smolt age and sea age as covariates. We then calculated the partial correlation coefficient to test the strength of association between freshwater and marine growth once the effects of freshwater and sea age had been statistically partialled out. This was achieved by calculating the correlation between the residuals of freshwater and marine growth after each had been regressed on freshwater and sea age.

Finally, in order to estimate the extent and magnitude of compensatory marine growth we computed the size rank of individual fish before and after migrating into the sea, and calculated Spearman rank correlation coefficients (*r*_s_) between freshwater and marine growth for each river. In the absence of compensatory marine growth, we would expect to find a positive correlation between smolt size and postsmolt growth, as larger than average smolts would continue to be larger than average at sea. On the other hand, if fish exhibited compensatory marine growth, we would expect to find no association between smolt size and postsmolt growth, as smaller than average fish would be able to catch-up (and move up the size rank) at sea.

## Results

### Reliability of Scale Measurements

There was no significant distortion of scale radius due to the impression process (*t*_29_ = 0.547, *P* = 0.465), indicating that acetate impressions gave an accurate, unbiased representation of scale size. Repeatabilities of scale size were high, both for smolt scale length (α-Cronbach = 0.879) and for scale size attained at the end of the first marine growing season (α-Cronbach = 0.918). Scale radius and fork length were positively correlated (*r* = +0.654, *P* = 0.001), and the relationship was not different among rivers (*F*_5,136_ = 1.002, *P* = 0.419) allowing us to use scale measurements to reconstruct changes in body size regardless of river identity.

Testing of interactions terms in ANCOVA indicated that the relationship between somatic growth and scale radius was not affected by age or body size (age×scale radius *F*_7,102_ = 0.983, *P* = 0.447; body size×scale radius *F*_14,91_ = 1.344, *P* = 0.197), i.e. slopes were homogeneous across age and size classes.

### Variability in Life Histories Among Populations

Sea trout populations differed significantly in sea age (*F*_5,132_ = 5.39, *P*<0.001), but not on smolt age (*F*_5,132_ = 0.55, *P* = 0.736). Populations did not vary in the size of smolts (*F*_5,126_ = 1.87, *P* = 0.104), once the overriding effect of smolt age (*F*_1,126_ = 110.53, *P*<0.001) had been statistically controlled for, but there was an interaction between smolt age and river of origin on smolt size (*F*_1,126_ = 110.53, *P* = 0.029) suggesting that different populations experienced different freshwater growth patterns before migrating to sea. Sea trout populations also differed in size of returning adults (*F*_5,126_ = 3.20, *P* = 0.009) once the important effect of sea age had been statistically accounted for (*F*_1,126_ = 25.51, *P*<0.001).

### Individual Variation in Reconstructed Growth Profiles

**Figure 2:**
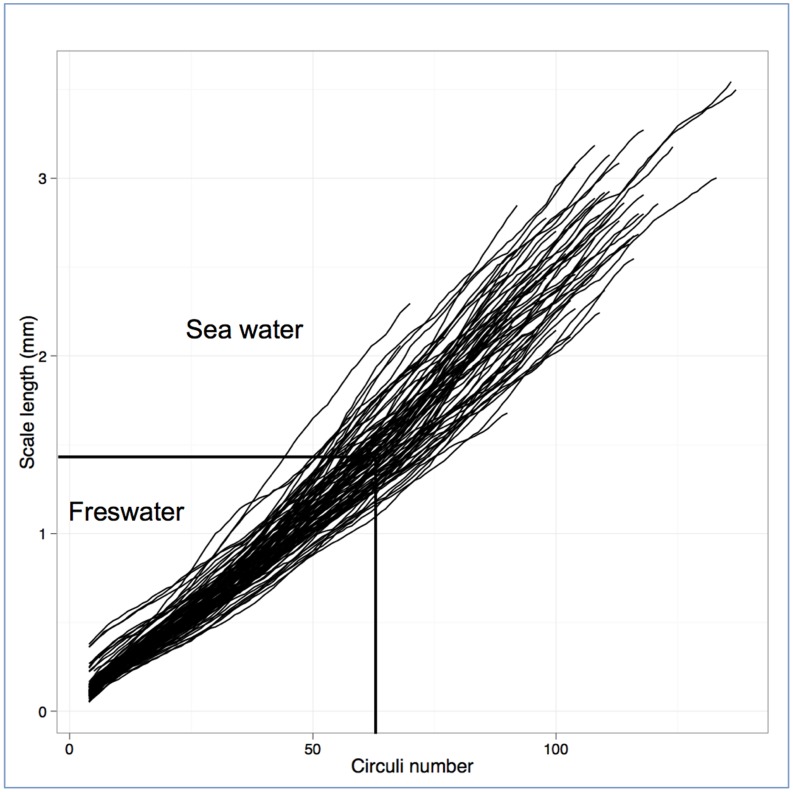
Individual scale growth profiles of migratory sea trout. Shown are estimated scale sizes (a proxy for body size) at each circuli number. Dark line represents mean values (95 CI) adjusted for a common smolt age and sea age at four key life stages (first winter in freshwater, entry into the sea – dotted line, end of first marine growing season, and adult returning to freshwater).

**Figure 3:**
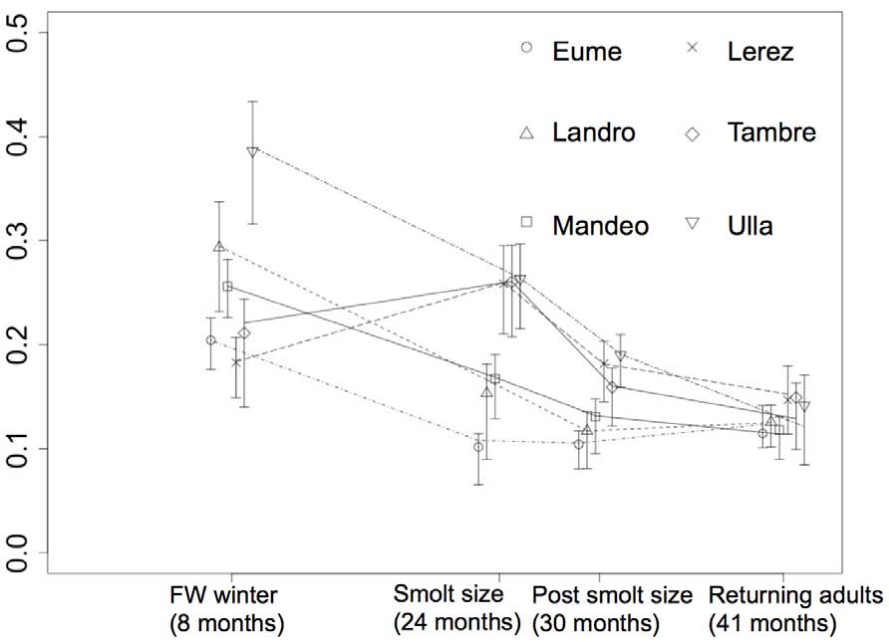
Temporal trends in the coefficient of variation (CV) for scale size (a proxy for body size) of three year old sea trout (2.1. age class) at four key life stages, stratified by population of origin. Shown are mean values and approximate 95% confidence intervals derived from 1,000 bootstrap replicates.

Individual variation in reconstructed growth profiles was high among individuals (Fig. 2; CV = 71.2%) and increased significantly over time (Fligner-Killen Test, χ^2^ = 59.91 df = 3, *P*<0.001) as fish followed diverging growth trajectories. The CV on body size, as inferred from variation in scale size and calculated for those returning adults that had spent two winters in freshwater and one winter at sea (the dominant age class), varied significantly among life stages (Fig. 3;Fligner-Killen test χ^2^ = 55.51, df = 3, *P*<0.001), being highest during the first 8 months of life (when juvenile mortality is highest) and lowest when adults returned from the sea. Populations differed significantly in CV for body size only during the first winter in freshwater (Fligner-Killen test χ^2^ = 14.50, df = 5, *P* = 0.013), but not at later stages (smolt χ^2^ = 5.33, df = 5, *P* = 0.376; PSG χ^2^ = 9.036, df = 5, *P* = 0.107; returning adults χ^2^ = 1.678, df = 5, *P* = 0.891).

### Compensatory Marine Growth

**Figure 4:**
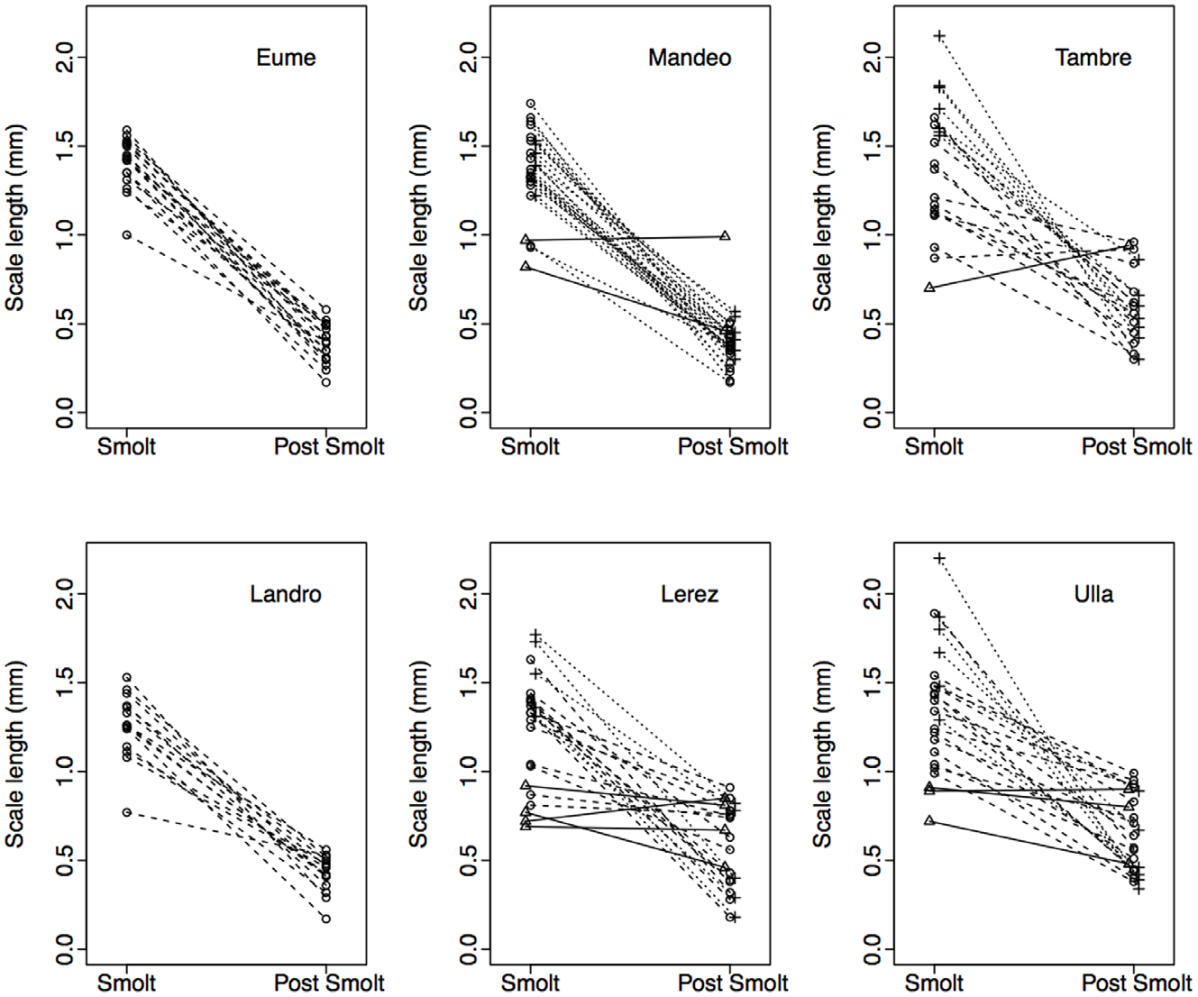
Individual growth reaction norms during the freshwater to marine transition in six sea trout populations, stratified by smolt age (○ 1 yr, ▵ 2 yr. +3 yr). Shown are matched comparisons between scale size at the moment of entry into the sea and subsequent scale growth increment during the first marine growing season (PSG) for each individual fish.

We concentrated on modelling post-smolt growth (PSG) during the first marine growing season, as this was the stage where there was greatest variation among individuals, and we could obtain data from all fish regardless of time spent at sea. We used as predictors the age and size of smolts, as well as the river of origin, to test the prediction that early growth performance during freshwater life was a good predictor of growth performance later in life at sea. Analysis of individual growth reaction norms (Fig. 4) indicated that smolt size at the moment of entry into the sea was negatively correlated with subsequent growth during the first marine growing season, once the effects of sea age and freshwater age had been statistically partialled out (partial correlation *r* = −0.233, df = 134, *P* = 0.006).

Following stepwise multiple regression, variation in post-smolt growth (PSG) was accounted for by river of origin (*F*_5,106_ = 11.079, *P*<0.001), smolt scale length (F_1,106_ = 7.838, *P*<0.001) and smolt age (F_2,106_ = 3.975, *P* = 0.048), in addition to a significant 3-way interaction among river, smolt age and smolt scale length (F_7,106_ = 2.967, *P* = 0.007). The minimal adequate model explained about 53% of the variance in PSG (*F*_31,106_ = 3.68, *P*<0.001, AIC = −474.72) and provided evidence of a negative relationship between size attained in freshwater and subsequent growth at sea.

Compensatory marine growth (revealed by the frequency of fish moving up the size rank following entry into the sea) was substantial and widespread. Thus, all populations exhibited compensatory growth, as suggested by non-significant rank correlation coefficients between smolt size and post-smolt growth (these ranged from *r*_s_ = −0.442, *P* = 0.051 in the R Tambre to *r*_s_ = 0.043, *P* = 0.846 for the R. Eume). The results also indicate that between 45% (R. Tambre) and 54% (R. Ulla) of fish displayed a gain in size rank at sea, depending on population of origin. Average change in post-migratory size rank of those fish displaying compensatory growth ranged from 7.6 positions in the R. Ulla to 10.4 positions in the R. Lerez, suggesting that size rank changes are likely to be biologically meaningful.

## Discussion

Our study on migratory trout indicates that there is a negative relationship between the size of juveniles in freshwater prior to migration and their subsequent growth at sea, once the effects of age on growth are controlled for. In general, a positive relationship between freshwater and marine growth is expected if marine food resources are patchily distributed, and dominant individuals can monopolize resources, as they tend to do in freshwater [43]. In contrast, when marine resources are evenly distributed, resource monopolization is not possible, or the costs of resource defence simply outweigh its benefits, a negative or no correlation between freshwater and marine growth can be expected [44].

Negative correlations between pre and post-migratory growth have been reported in many studies of anadromous salmonids and suggest the existence of trade-offs, whereby traits that promote fast growth in one environment do not translate into rapid growth in other environments. Indeed, analysis of 61 datasets representing four salmonid species (Table 2) indicates that there is no significant association between smolt size and marine growth in the majority of studies (55.7%), and that negative relationships (29.5% of cases) tend to be more likely to occur than positive ones (14.8% of comparisons), though not statistically so (χ^2^ = 3.0 df = 1, *P* = 0.083). Our study, like most other studies, suggests that juvenile size is a poor predictor of subsequent growth at sea because individuals that grow slowly in freshwater are able to compensate with enhanced post-migratory growth later in life. There are various possible reasons for this.

**Table 2:** Studies on anadromous salmonids investigating the relationship between smolt size and post-smolt growth.

Relationship between smolt sizeand post-smolt growth							Type ofFish	
Species	Location	−	NS	+	Period	Method		Reference
*S. salar*	Matre Aq St.	0	0	1	1981	MR	H	[27]
*S. salar*	R. Narcea	5	1	0	1986–1990	BC	W	[16]
*S. salar*	R. Esva	3	2	0	1986–1990	BC	W	[16]
*S. salar*	R. Cares	1	1	0	1987–1988	BC	W	[16]
*S. salar*	R. Penobscot	0	0	1	1973–1990	SG	H	[18]
*S. salar*	Finland	0	0	1	1980–1991	MR	H	[26]
*S. salar*	R. Imsa	1	0	0	1981–2003	MR	H	[23]
*S. salar*	R. Alta	1	0	0	1993–1995	MR	W	[22]
*S. salar*	Gulf St. Lawrence	0	1	0	1982–1984	SG	W	[25]
*S. salar*	R. Asón	1	0	0	1948–2003	BC	W	[33]
*S. salar*	R. Miramichi	0	1	0	1956–2003	BC	W	[87]
*S. trutta*	Finland	1	0	0	1915–1989	Cline	W	[14]
*O. kisutch*	Oregon	0	9	2	1991–2000	BC	W	[44]
*O. kisutch*	Washington	0	8	2	1991–2000	BC	W	[44]
*O. kisutch*	British Col.	2	9	0	1991–2000	BC	W	[44]
*O. kisutch*	British Col.	1	0	0	1990	MR	H	[88]
*O. mykiss*	British Col.	0	0	1	1990	MR	H	[88]
*S. laucomaenis*	R. Nairo & Haraki	1	2	1	1990–1991	BC	W	[24]
*S. trutta*	6 rivers, NW Spain	1	0	0	2002	SG	W	This study
								
	Total	18	34	9				

Studies that found a negative relationship (−), no significant relationship (NS), and a positive relationship (+) are indicated. Method: MR mark and recapture, BC back-calculation of juvenile size, SG scale growth. Type of fish : H hatchery, W wild.

Firstly, it is possible that gender differences (which were not measured in our study) may introduce a source of variation in the relationship between pre- and post-migratory body size, for example if males and females achieve different sizes [45] or are under different selection pressures [46]–[47]. However, studies where gender has been controlled for [22] failed to find a positive relationship between freshwater size and marine growth, or found an inverse relationship, suggesting that the lack of association is a common phenomenon. Lack of association between pre- and post-migratory growth may also be due to low statistical power. For example, with our sample size (n = 138) we were able to detect a correlation greater than 0.21 or lower than −0.21 with 80% power, but power to detect weaker associations (and to reject the null hypothesis of no correlation) may have been too low in previous studies [48].

Thirdly, random measurement error would tend to blur any relationship between freshwater and marine growth. This may be particularly true if back-calculation of body size (instead of scale growth) is used because *in situ* measurements of fish size may lack precision in the field [49]. On the other hand, scale size measured in the laboratory has been shown to be a reliable indicator of smolt size in salmonids [50], and our study shows that repeatability in scale size was high and that no bias due to the scale impression process could be detected. Therefore, we are confident that our estimates of scale growth are reliable, and that these allow us to reconstruct changes in growth of migratory trout and to compare growth trajectories of individuals.

Using size comparisons to infer rate of growth implicitly assumes that individuals have grown over the same period of time. This would be true only if all fish had emerged and smolted at the same time, and grown over the same length of time in the marine environment. Otherwise, variation in growth rates may be confounded by variation in the length of the growing season, and this may mask the detection of size trade-offs. All adults used in our study were caught in freshwater over a relatively short period of time (August–October), and we excluded from analysis those fish that had spent less than one full winter at sea to reduce additional sources of variation. Information on timing of alevin emergence was not available in our study, but development in southern brown trout populations is rapid and emergence is likely to be short and less protracted than in more northern latitudes [51]–[52]. Data from a downstream smolt trap in one of the study rivers (R. Ulla) indicates that the timing of smolt migration is highly clumped, with 50% of smolts moving downstream over a relatively narrow time window (average during 1998–2001 was 16 days, range = 6–28 days). This suggests that the observed variation in the size of individuals cannot solely be explained by differences in the timing of emergence, timing of smolting, or in length of the growing season, which are thought to have been similar among individuals in our study. Other studies have also shown that such differences are small in relation to variation in smolt size and post-smolt growth [22].

Compensatory growth, where individuals that grow poorly during periods of nutritional deficit are then able to accelerate their growth and “catch-up” when conditions improve [10], [53], is the most plausible explanation for the observed inverse relationship between freshwater and marine growth shown in our study. Migration has been viewed as a strategy to “escape” from harsh conditions, typically caused by predation and competition from increasingly larger conspecifics [54]. Among facultative anadromous salmonids, migration is thought to represent a trade-off between better growth opportunities at sea, but also greater risk from predation [7], [55]. Although there is some evidence for density-dependence in salmonid marine survival [56], the evidence is not compelling. In contrast, evidence of density-dependent marine growth is much more common [57]–[59], though this is most readily apparent during the late marine phase, presumably because the costs of reduced growth are less likely to have an impact on survival later in life [60]. Often the mean scale radius of salmonid migrants is significantly smaller than that of returning adults from the same cohort, suggesting that small migrants sustain high mortality at sea [49]. More generally, large individuals often have a survival advantage over small conspecifics, both in freshwater and in the sea [50], [61]–[64] adding some support to the ‘bigger is better’ hypothesis. However, there are also many cases when no such size advantage is apparent [65], or when size-selective mortality favours a large body size in some years and a small size in others [66]–[67], perhaps because phenotypic adjustment is the norm in salmonid populations [15], [68].

Whatever the precise direction of selection, a recent meta-analysis [69] has shown that fish are subjected to extreme selection on body size during early life, the strength of which typically decreases over time. This is consistent with our results on migratory trout, which indicate that the CV for body size (as inferred from variation in scale size) varies markedly over the life time of individuals, decreasing with time as fish migrated from freshwater into the sea. With the exception of the first freshwater winter, no differences were found among six neighbouring populations, suggesting that once the critical time for survival has passed [70]–[71], selection probably operates in a similar way in neighbouring rivers.

The coefficient of variation (CV) is useful for quantifying phenotypic variation [28] and for examining ontogenetic size changes in longitudinal studies [51]. CV is expected to decrease when stabilizing selection acts upon a continuous trait, and can be useful as a preliminary step towards more detailed selection analysis [72]. Individual variation in growth rates decreases with increasing competition in brown trout [29], [46], suggesting that changes in the CV could track changes in selection intensity. During the first stages of their lives, brown trout juveniles tend to be subjected to strong selection mediated by both density-dependent and density-independent processes [73], the relative strengths of which may differ markedly from site to site, and also from year to year [70], [74]–[75]. Early density dependent processes are thought to decrease at smolting, when territoriality in migratory salmonids disappears and the strength of intra-specific competition weakens in preparation for the marine migration [55]. Osmoregulation amongs smolts is accompanied by tissue differentiation of gut, gill and kidney [55], [76], and juveniles must reach a minimum threshold smolt size or will not smolt [55]. Smolt size, not age, is the primary determinant of marine survival in anadromous salmonids [76], and strong selection for smolt size may therefore be expected to exist. Indeed, our study indicates that there was relatively little variation for smolt size in migratory brown trout, and a significant decrease in CV from the first freshwater winter onwards. Individual variation in salmonids body size has been shown to decrease after periods of intense selection, for example following size-selective predation [77]–[78] or poor feeding conditions at sea [61].

Some authors have found that variation in smolt size and age decrease with increasing stream size (e.g. [79]), apparently because the success of large juveniles is more variable and less predictable in small than in large streams [46]. However, no such relationship was apparent in our study populations, which displayed the same narrow variation in smolt size in spite of relatively large differences in stream size and in demographic parameters (Table 1), again suggesting that smolt size is probably under strong selection.

In summary, our study indicates that there is an inverse relationship between pre- and post-migratory size in migratory trout, which we interpret as indicative of marine compensatory growth. The CV on body size was highest during the first freshwater winter and decreased during the marine phase, and this appears to track changes in juvenile mortality. In addition to heritable variation, phenotypic plasticity and genotype×environment interactions, ontogenetic variation due to changes in the timing or rate of developmental events (growth heterochrony [80]) can be an important source of body size variation [81]–[82]. This is particularly true in fishes, which have indeterminate growth, and where even small changes in heterochrony can result in large morphological differences among individuals [83]–[84]). Gene duplication may have also allowed large phenotypic diversification amongst the teleosts [85], as it provides “extra genetic material freed from the need to function in only one way, and therefore available for experimental [evolutionary] change” [81].

We suggest that despite a widespread selective advantage of large body size in freshwater, freshwater growth is a poor predictor of final body size amongst migratory fish because selection may favour growth heterochrony leading to marine compensatory growth. Marine compensatory growth allows size-depressed individuals to catch-up later in life and may, therefore, negate any initial size advantage acquired in freshwater. Such a mechanism could be responsible for the heterogeneity in growth trajectories observed in our study, a pattern not readily detected in captivity [86], where food is generally plentiful and natural selection relaxed. Ultimately, growth heterochrony could help maintain phenotypic variation in sea trout because anadromous individuals could attain similar sizes at spawning (and hence have similar fecundities) despite having experienced very different growth trajectories early in life.
